# Effects of preoperative sarcopenia-related parameters on the musculoskeletal and metabolic outcomes after bariatric surgery: a one-year longitudinal study in females

**DOI:** 10.1038/s41598-023-40681-w

**Published:** 2023-08-17

**Authors:** Nara Nóbrega Crispim Carvalho, Vinícius José Baccin Martins, João Modesto Filho, Adélia da Costa Pereira de Arruda Neta, Flávia Cristina Fernandes Pimenta, José Luiz de Brito Alves

**Affiliations:** 1 Department of Nutrition, Health Sciences Center, Federal University of Paraiba, Campus I – Jd. Cidade Universitária, Joao Pessoa, PB 58051-900 Brazil; 2 Department of Endocrinology, Lauro Wanderley University Hospital, Federal University of Paraiba, Joao Pessoa, Brazil; 3 Lauro Wanderley Hospital, Federal University of Paraiba, Joao Pessoa, Brazil

**Keywords:** Medical research, Nutrition disorders

## Abstract

Reduced muscle mass and/or strength are risk factors for metabolic and musculoskeletal impairment. The present study evaluated anthropometric, metabolic, and musculoskeletal outcomes in females with and without sarcopenic-obesity parameters who underwent bariatric surgery during a 1-year follow-up. A prospective, single-center cohort study was conducted in females with obesity undergoing preoperative evaluation for surgery. In the preoperative period, females were allocated into obesity with sarcopenic-obesity parameters (SOP group, n = 15) and without sarcopenic-obesity parameters (obesity group, n = 21). Sarcopenic obesity parameters were defined as lower appendicular skeletal mass adjusted for weight (ASM/wt) and/or low handgrip strength (HGS). Anthropometric, metabolic, and musculoskeletal parameters were assessed before surgery and at 3 months, 6 months, and a 1-year after bariatric surgery. Weight loss was similar between groups (*p* > 0.05). Weight, body mass index, fat mass, body fat percentage, skeletal muscle mass, fat-free mass, fat-free mass index, HGS were reduced in both groups during the 1-year follow-up (*p* < 0.05). However, when muscle mass and strength were analyzed relative to body size, an improvement after bariatric surgery was found in both groups (p < 0.05). Total cholesterol, LDL-c, triglycerides, fasting glucose, glycated hemoglobin, insulin, and insulin resistance were reduced in both groups during the 1-year follow-up (*p* < 0.05). In addition, HDL-c serum concentration increased in females with and without sarcopenic-obesity parameters over the 1-year follow-up (*p* < 0.05). Both groups had decreased bone mineral density (BMD) at all sites (lumbar spine, femoral neck, and total femur) over the 1-year follow-up (*p* < 0.05). The highest quartile of ASM/wt was positively associated with BMD variables in a longitudinal analysis, suggesting that preserved ASM/wt in pre-surgery may be beneficial for BMD after 1 year of bariatric surgery. The results showed that bariatric surgery promotes similar musculoskeletal and metabolic changes in females with preserved muscle mass and strength or in females with sarcopenia-related parameters.

## Introduction

Surgical treatment for severe obesity has increased worldwide due to the increasing prevalence of obesity. Bariatric surgery is an effective treatment option for weight loss, improvement of comorbidities, and reduction of mortality^1^. Although several health benefits have been reported after bariatric surgery, many individuals do not experience the expected weight loss and improvement or remission of comorbidities, likely due to patients’ clinical conditions before surgery, such as age and comorbidities, and associated genetic factors^2,3^.

Classically, the preoperative evaluation for bariatric surgery is performed by an interprofessional team and includes the assessment of psychosocial factors, anthropometric and nutritional variables, complete screening for cardiovascular disease and obstructive sleep apnea, and a comprehensive metabolic panel^4^. However, it is important to emphasize that although obesity affects other systems, such as the musculoskeletal system, musculoskeletal evaluation is not a routinely recommended procedure in the workup for bariatric surgery.

Appendicular skeletal mass adjusted for weight (ASM/wt) in females with obesity was positively associated with handgrip strength (HGS) and bone mineral density (BMD)^5^. Bariatric surgery results in changes in body composition with loss of fat mass, skeletal muscle, and BMD^6,7^. A meta-analysis showed that individuals who underwent bariatric surgery lost 8 kg of lean body mass within 1 year of surgery^8^. Identifying patients at high risk of excessive muscle loss may help to develop strategies to limit muscle loss and complications after bariatric surgery^9^.

Sarcopenia-related parameters combined with high adiposity is a risk factor for several complications, including physical disability, falls, osteoporosis, fractures, cardiovascular and metabolic complications, and mortality risk^10–14^. Sarcopenia is strongly associated with advancing age, with 1 to 2% of skeletal muscle mass and 1.5 to 5% of muscle strength lost annually after age 50. However, regardless of age, low-grade chronic inflammation promoted by obesity is a risk factor for musculoskeletal disability and sarcopenia^15^.

Considering that the lack of studies investigating whether preoperative sarcopenic-obesity parameters disrupt the musculoskeletal and metabolic outcomes of bariatric surgery, this study evaluated anthropometric, metabolic, and musculoskeletal outcomes in females with low muscle mass and/or strength who underwent bariatric surgery for a 1-year follow-up. The hypothesis tested is that females with reduced muscle mass and/or strength before bariatric surgery have worse metabolic and musculoskeletal outcomes during a 1-year follow-up compared to females with only obesity alone.

## Materials and methods

### Ethical aspects

This study was conducted in accordance with the Declaration of Helsinki. The study was approved by the Research Ethics Committee of Lauro Wanderley University Hospital, Federal University of Paraiba (Reference number 2.548.555). All patients gave written informed consent. All procedures were conducted in agreement with the Resolution 466/2012 of the National Health Council and the International.

### Design and subjects

Seventy-five participants were evaluated (convenience sample) before bariatric surgery; 44 participants underwent surgery. Male were excluded from the sample due to the small number of participants (n = 4). In addition, four females were lost to follow-up. Females with obesity, aged 18–60 years, with a body mass index ≥ 40 kg/m^2^ or ≥ 35 kg/m^2^ with comorbidities, previously referred to the bariatric surgery service of the Lauro Wanderley University Hospital were included in the study. This hospital is the only one in the State of Paraíba accredited by the public health system to perform bariatric surgery (sleeve gastrectomy or Roux-en-Y gastric bypass). Of these females, 9 underwent bariatric surgery using the sleeve gastrectomy and 27 underwent surgery using the Roux-in-Y gastric bypass surgery.

Participants were recruited and then divided into females with obesity and sarcopenic-obesity parameters (SOP group) and females with obesity (obesity group). Sarcopenic-obesity parameters were defined as low ASM/wt and/or HGS in the lowest quartile of the sample. All females had a high percentage of body fat. In addition, all participants had well-controlled comorbidities, were taking medications regularly, and had stable weight after dietary monitoring.

The occurrence of arrhythmias, cardiac transplantation, cardiac pacemakers, ischemic and non-ischemic cardiomyopathy, psychiatric disorders, and malignant neoplasms was used as exclusion criteria. Participants with neurological, osteoarticular, hepatic, pulmonary, and renal dysfunction were also excluded. The females underwent anthropometric, metabolic and body composition, and bone mass assessments before surgery and at 3, 6, and 12 months after bariatric surgery.

### Clinical, anthropometric, and blood pressure measurements

Two questionnaires were administered to participants to collect information before and after bariatric surgery. The first questionnaire collected socio-demographic data, medical history (previous diseases, menopausal history, history of atraumatic bone fracture, duration of illness, and use of medications), and lifestyle (physical activity, dietary counseling, and smoking). The time of diagnosis of type 2 diabetes mellitus and hypertension was self-reported by the participants. The second questionnaire collected data on the type of surgery performed, surgical complications, and medications in use.

Participants were weighed in light clothing, barefoot, using a scale with an accuracy of 0.1 kg (Inbody 370). Height was measured to the nearest 0.5 cm using a stadiometer (Caumaq), and body mass index was calculated by dividing weight in kilograms by height in meters squared.

Calf and neck circumferences were measured using an inelastic tape. Calf circumference was measured in a sitting position, perpendicular to the long axis of the calf, by moving the tape up and down until the maximum circumference was found. Neck circumference was measured from the midpoint of the cervical spine to the anterior center of the neck. Weight loss was measured by subtracting the total weight measured at 3, 6, and 12 months from the baseline weight. Blood pressure was taken in the morning (8–11 am) in a quiet room, according to early studies^16,17^.

### Body composition assessment

Bioelectrical impedance analysis (Inbody 370, Model JMW140, Chungcheongnam-do, KOREA), eight-point tactile electrodes, and multi-frequency (5 kHz, 50 kHz, 250 kHz) was used to assess body composition. It was recommended fast for 12 h, not to do strenuous physical exercises and not to be in the menstrual period. Fat mass (kg) and skeletal muscle mass (kg) of all body segments (arms, legs, and trunk), as well as fat-free mass and body fat percentage, were obtained from the manufacturer’s algorithm, using sex, age, weight, and height.

Appendicular skeletal mass (kg) was obtained by summing the skeletal muscle mass of both arms and legs. The following indices were calculated: muscle mass index (ASM/wt) and fat-free mass index (fat-free mass adjusted for height squared).

BMD, T-score, and Z-score at the lumbar spine (L1-L4), femoral neck, and total femur were assessed by dual-energy X-ray absorptiometry (DXA) using a properly calibrated densitometer (model Lunar 8743, Medical Systems Lunar, Madison, USA). DXA composition data were not used because the exam was not performed properly for whole body composition.

### Physical function

HGS was measured in kilograms using a Jamar digital dynamometer (Sammons Preston Inc., IL, USA). Three measurements were taken in each hand, and the mean values of these three measurements in the dominant hand was used as the final value. The examination was performed with a 30-s rest between measurements^18^.

The six-minute walk (6MWT) test was used to measure physical performance. The space was demarcated every meters to facilitate the calculation of the distance covered. The gait speed was then calculated using the formula: speed (m/s) = distance covered in meters/360 seconds^19^. The test was performed on a flat surface in a closed, air-conditioned environment^20^.

### Biochemical measurements

Blood samples were collected after a 12-h fast and without strenuous exercise for the previous 24 h. Fasting glucose, triglycerides, cholesterol, and high-density lipoprotein cholesterol (HDL-c) were measured using an automated enzymatic method (Autoanalyzer; Technicon, Tarrytown, NY, USA). Low-density lipoprotein cholesterol (LDL-c) was calculated using the Friedwald formula^21^. Insulin was determined by chemiluminescence immunoassay. Glycated hemoglobin (HbA1c) was determined by high-performance liquid chromatography, and high-sensitivity quantitative C-reactive protein (hs-CRP) was quantified by turbidimetry. Homeostasis model assessment insulin resistance (HOMA-IR) was used to measure insulin resistance and was calculated as fasting insulin (uU/L) x fasting glucose (mg/dL) divided by 22.5.

### Statistical analysis

Baseline data and percent weight loss were analyzed by independent *t* test, Mann–Whitney, or chi-squared. Body composition, muscle function, biochemical variables, and bone mass were analyzed by mixed between-within-subjects ANOVA. Generalized estimating equations (GEE) models were used to prospectively examine the association between ASM/wt or HGS and BMD. The bone mineral density variables were used for the GEE models with normal distribution using the “Gaussian family” specification. Potential confounders included in the analysis were: age, body mass index, body fat, and HOMA-IR. Statistical analyses were performed using SPSS 20.0 (IBM Corp., Armonk, NY, USA), and differences between groups were considered statistically significant when p-value < 0.05. Missing values at three (n = 8 participants) and six (n = 7 participants) months were imputed using the mean of the variable.

## Results

### Baseline

Baseline characteristics regarding age, anthropometric measurements, blood pressure, and history of disease were similar between groups (Table 1). At baseline, females with SOP had reduced ASM/wt, total skeletal muscle mass, HGS, and HGS adjusted for body mass index, gait speed, L1-L4 BMD, femoral neck BMD, and HDL cholesterol, and higher HbA1c compared with females with obesity alone (Table 1). No differences were found in the rates of surgical Roux-in-Y gastric bypass and gastric sleeve surgery (*p* > 0.05, Table 1).

**Table 1:** Baseline characteristics and surgery in females with obesity and sarcopenic-obesity parameters.

Variables	Obesity (n = 21)	Sarcopenic-obesity parameters (SOP, n = 15)	*p*-value
Age (years)	40.4 ± 8.5	39.0 ± 11.2	0.672
Body mass index (kg/m^2^)	41.5 ± 4.6	44.0 ± 4.4	0.131
ASM/wt	21.1 ± 1.8	18.6 ± 1.8	< 0.001
Handgrip strength (HGS, kg)	32.5 ± 4.8	23.5 ± 3.8	< 0.001
HGS/body mass index	0.79 ± 0.1	0.53 ± 0.09	< 0.001
Neck circumference (cm)	37.9 ± 3.2	39.2 ± 2.4	0.233
Calf circumference (cm)	44.9 ± 4.8	42.7 ± 4.1	0.288
Total skeletal muscle mass (kg)	46.6 ± 5.4	41.7 ± 5.7	0.013
Total skeletal muscle mass/BMI	1.1 ± 0.1	0.9 ± 0.1	< 0.001
Total fat mass (kg)	50.5 ± 14.0	55.1 ± 10.2	0.289
Fat-free mass index (kg/m^2^)	19.8 ± 1.9	20.4 ± 1.8	0.346
Gait speed (m/s)	1.1 ± 0.15	0.97 ± 0.16	0.017
L1-L4 BMD (g/cm^2^)	1.27 ± 0.15	1.15 ± 0.12	0.014
Femoral neck BMD (g/cm^2^)	1.12 ± 0.11	1.03 ± 0.12	0.025
Total femur BMD (g/cm^2^)	1.17 ± 0.14	1.09 ± 0.15	0.110
Total cholesterol (mg/dL)	176 ± 25	194 ± 38	0.095
HDL-cholesterol (mg/dL)	53 ± 10	46 ± 9	0.038
LDL-cholesterol (mg/dL)	99 ± 22	116 ± 32	0.067
Triglycerides (mg/dL)^#^	127 (34–443)	152 (62–369)	0.421
Insulin (µU/mL)^#^	18 (4–67)	18 (9–33)	0.452
Fasting glucose (mg/dL)^#^	91 (67–172)	95 (62–137)	0.207
HOMA-IR^#^	3.8 (0.9–7.3)	4.6 (1.5–10.3)	0.382
HbA1c (%)^#^	5.9 (5.3–9.8)	6.1 (5.3–8.1)	0.045
hs-CRP (mg/L)^#^	6.0 (0.5–19.2)	9.1 (1.2–46.3)	0.576
Systolic blood pressure (mmHg)	112 ± 13	110 ± 15	0.764
Diastolic blood pressure (mmHg)	74 ± 9	72 ± 7	0.502
Type 2 diabetes mellitus (% (n))	14.3 (3)	35.7 (5)	0.285
Hypertension (% (n))	52.4 (11)	57.1 (8)	0.944
Sleeve gastrectomy—% (n)	23.8 (5)	26.6 (4)	0.990
Roux-en-Y gastric bypass—% (n)	76.2 (16)	73.4 (11)	0.990

Data expressed in mean ± SD, median (min–max) or % (n). Independent *t* test, Mann–Whitney^#^, or chi-square test was used.

*ASM/wt* appendicular skeletal mass adjusted for weight, *HDL*- *cholesterol* high density lipoprotein cholesterol, *LDL*-*cholesterol* low density lipoprotein cholesterol, *HOMA*-*IR* homeostasis model assessment—insulin resistance, *HbA1c* glycated hemoglobin, *hs*-*CRP* high-sensitive c-reactive protein.

### Follow-up after bariatric surgery

#### Weight loss, body composition, and muscle function after bariatric surgery

Both groups significantly decreased weight, body mass index, fat mass, body fat percentage, skeletal muscle mass, fat-free mass, fat-free mass index, gait speed, and HGS during the 1-year follow-up (*p* < 0.05, Table 2). The percentage of weight loss after 1 year of bariatric surgery was similar between groups (obesity: 24.3 ± 11.5 vs. sarcopenic-obesity parameters: 31.0 ± 9.9%, *p* = 0.09). Females in both groups showed increased ASM/wt, HGS adjusted for body mass index and gait speed during a 1-year follow-up (*p* < 0.05, Table 2). Although females with SOP had lower fat-free mass, lower skeletal muscle mass, lower ASM/wt, lower HGS, and lower HGS adjusted for body mass index in a cross-sectional comparison (*p* < 0.05, Table 2), no difference was found for the time x group interaction for these variables (*I* > 0.05, Table 2).

**Table 2:** Effects of bariatric surgery on body composition and muscle function variables in females with obesity and sarcopenic-obesity parameters over a 1-year follow-up.

	Obesity (n = 21)				Sarcopenic-obesity parameters (SOP, n = 15)				*p*-value time	*p*-value group	*I*
	Baseline	3 months	6 months	1 year	Baseline	3 months	6 months	1 year			
Weight (kg)	109.8 ± 13.9	91.2 ± 12.6	86.9 ± 12.4	82.7 ± 13.8	106.5 ± 11.1	86.8 ± 11.2	80.7 ± 10.6	73.1 ± 10.2	< 0.001	0.125	0.483
Body mass index (kg/m^2^)	41.5 ± 4.6	34.7 ± 3.9	32.2 ± 4.1	30.6 ± 4.9	44.0 ± 4.4	37.0 ± 4.1	33.2 ± 4.3	29.6 ± 4.3	< 0.001	0.377	0.060
Total skeletal muscle mass (kg)	46.6 ± 5.4	43.8 ± 3.9	42.8 ± 3.5	42.1 ± 3.9	41.7 ± 5.7	39.3 ± 4.5	38.1 ± 4.5	36.8 ± 4.2	< 0.001	0.002	0.403
Total skeletal muscle mass/body mass index	1.1 ± 0.1	1.3 ± 0.1	1.3 ± 0.2	1.4 ± 0.2	0.9 ± 0.1	1.1 ± 0.1	1.2 ± 0.2	1.3 ± 0.2	< 0.001	0.002	0.277
Trunk muscle mass (kg)	23.2 ± 2.5	21.1 ± 2.7	21.2 ± 2.1	20.8 ± 2.2	23.8 ± 3.5	21.0 ± 4.2	21.0 ± 4.0	20.7 ± 4.3	< 0.001	0.974	0.886
ASM/wt	21.1 ± 1.8	22.4 ± 1.2	23.9 ± 1.8	24.7 ± 2.3	18.6 ± 1.8	21.2 ± 1.8	22.2 ± 2.6	24 ± 3.5	< 0.001	0.025	0.066
Fat-free mass (kg)	48.7 ± 4.4	45.7 ± 3.7	46.0 ± 3.7	45.9 ± 3.6	41.7 ± 9.9	39.7 ± 8.4	39.5 ± 8.5	39.4 ± 8.4	0.005	0.007	0.773
Fat-free mass index (kg/m^2^)	19.8 ± 1.9	19.1 ± 1.7	19.0 ± 1.7	18.7 ± 1.9	20.4 ± 1.8	18.9 ± 1.7	18.3 ± 1.5	18.1 ± 1.8	< 0.001	0.663	0.120
Total fat mass (kg)	50.5 ± 14.0	42.1 ± 8.8	35.2 ± 8.8	32.1 ± 10.4	55.1 ± 10.2	43.0 ± 6.2	35.9 ± 7.9	26.7 ± 9.5	< 0.001	0.086	0.952
Trunk fat mass (kg)	26.1 ± 4.1	21.4 ± 2.9	19.6 ± 4.2	17.1 ± 5.6	29.3 ± 5.1	24.6 ± 5.2	22.6 ± 6.2	19.6 ± 8.0	< 0.001	0.062	0.977
Body fat (%)	49.5 ± 5.1	45.9 ± 3.7	41.1 ± 5.5	39.2 ± 7.1	53.3 ± 4.9	48.6 ± 3.4	44.1 ± 6.3	38.0 ± 9.5	< 0.001	0.204	0.134
Handgrip strength (HGS, kg)	32.5 ± 4.8	30.9 ± 3.5	30.0 ± 3.0	28.2 ± 4.3	23.5 ± 3.8	22.5 ± 4.2	22.6 ± 4.4	21.4 ± 3.6	0.002	< 0.001	0.400
HGS/body mass index	0.79 ± 0.15	0.90 ± 0.13	0.95 ± 0.12	0.95 ± 0.20	0.53 ± 0.09	0.61 ± 0.12	0.69 ± 0.17	0.74 ± 0.22	< 0.001	< 0.001	0.329
Gait speed (m/s)	1.1 ± 0.15	1.17 ± 0.13	1.17 ± 0.15	1.18 ± 0.17	0.97 ± 0.16	1.10 ± 0.11	1.11 ± 0.15	1.13 ± 0.11	0.010	0.070	0.476

Mixed between-within-subjects ANOVA. Data are expressed as mean ± SD.

*I* interaction, *ASM*/*wt* appendicular skeletal mass adjusted for weight, *HGS* Handgrip strength.

Both groups showed reduced BMD at all sites (lumbar spine, femoral neck, and total femur) during a 1-year follow-up period (*p* < 0.05, Table 3). Although females with SOP had lower BMD, Z-score, and T-score at the lumbar spine and femoral neck in a cross-sectional comparison (*p* < 0.05, Table 3), no difference was found for the time x group interaction for these variables (*I* > 0.05, Table 3).

**Table 3:** Effects of bariatric surgery on bone mineral density variables in females with obesity and with sarcopenic-obesity parameters over a 1-year follow-up.

BMD variables	Obesity (n = 21)				Sarcopenic-obesity parameters (SOP, n = 15)				*p-*value time	*p-*value group	*I*
	Baseline	3 months	6 months	1 year	Baseline	3 months	6 months	1 year			
L1-L4 BMD (g/cm^2^)	1.27 ± 0.15	1.27 ± 0.16	1.25 ± 0.16	1.22 ± 0.15	1.15 ± 0.12	1.16 ± 0.13	1.14 ± 0.12	1.11 ± 0.13	0.001	0.031	0.730
L1-L4 T-score	0.68 ± 1.26	0.69 ± 1.29	0.54 ± 1.27	0.26 ± 1.22	− 0.41 ± 1.12	− 0.25 ± 1.09	− 0.35 ± 1.01	− 0.58 ± 1.11	0.001	0.028	0.530
L1-L4 Z-score	0.93 ± 1.15	0.83 ± 1.29	0.71 ± 1.26	0.46 ± 1.13	− 0.13 ± 0.94	− 0.01 ± 0.98	− 0.05 ± 0.93	− 0.32 ± 1.00	0.002	0.030	0.451
Femoral neck BMD (g/cm^2^)	1.12 ± 0.11	1.10 ± 0.11	1.09 ± 0.11	1.06 ± 0.10	1.03 ± 0.12	1.02 ± 0.13	1.00 ± 0.13	0.99 ± 0.13	< 0.001	0.052	0.897
Femoral neck T-score	0.60 ± 0.83	0.51 ± 0.80	0.41 ± 0.80	0.23 ± 0.72	− 0.11 ± 0.89	− 0.20 ± 0.94	− 0.28 ± 0.96	− 0.38 ± 0.96	< 0.001	0.030	0.823
Femoral neck Z-score	1.11 ± 0.84	0.98 ± 0.84	0.93 ± 0.81	0.76 ± 0.69	0.47 ± 0.72	0.42 ± 0.75	0.38 ± 0.74	0.25 ± 0.77	< 0.001	0.047	0.558
Total femur BMD (g/cm^2^)	1.17 ± 0.14	1.15 ± 0.14	1.14 ± 0.14	1.11 ± 0.13	1.09 ± 0.15	1.08 ± 0.16	1.07 ± 0.15	1.06 ± 0.16	< 0.001	0.233	0.316
Total femur T-score	1.32 ± 1.14	1.16 ± 1.15	1.06 ± 1.12	0.84 ± 1.09	0.72 ± 1.24	0.70 ± 1.28	0.58 ± 1.23	0.52 ± 1.24	< 0.001	0.245	0.154
Total femur Z-score	1.60 ± 1.14	1.47 ± 1.15	1.36 ± 1.11	1.17 ± 1.04	1.01 ± 1.17	0.98 ± 1.20	0.87 ± 1.14	0.79 ± 1.17	< 0.001	0.231	0.412

Mixed between-within-subjects ANOVA. Data are expressed as mean ± SD.

*BMD* bone mineral density, *L1*–*L4* lumbar spine from 1 to 4.

#### Association between sarcopenia parameters and bone mineral density throughout follow-up

The highest quartile of ASM/wt was positively associated with L1-L4 BMD, femoral neck BMD, and femur BMD in a crude analysis and in models adjusted for age, body mass index, body fat percentage, and HOMA-IR (Table 4). On the other hand, L1-L4 BMD, femoral neck and total femur BMD were not associated with HGS over time (Table 4).

**Table 4:** Association over follow-up between low muscle mass or low muscle strength with bone mineral density.

	L1-L4 BMD				L1-L4 (T-score)				L1-L4 (Z-score)			
	ASM/wt (highest quartile) β	*p*	HGS (highest quartile) β	*p*	ASM/wt (highest quartile) β	*p*	HGS (highest quartile) β	*p*	ASM/wt (highest quartile) β	*p*	HGS (highest quartile) β	*p*
Model 1	0.156	**0.028**	0.092	0.094	1.257	**0.029**	0.856	0.080	1.058	**0.037**	0.501	0.255
Model 2	0.157	**0.019**	0.088	0.099	1.270	**0.021**	0.830	0.088	1.043	**0.048**	0.530	0.252
Model 3	0.263	**0.005**	0.075	0.195	2.091	**0.007**	0.713	0.165	1.925	**0.012**	0.433	0.393
Model 4	0.263	**0.004**	0.072	0.217	2.081	**0.005**	0.686	0.186	1.912	**0.009**	0.389	0.454

	Femoral neck BMD				Femoral neck (T-score)				Femoral neck (Z-score)			
	ASM/wt (highest quartile) β	*p*	HGS (highest quartile) β	*p*	ASM/wt (highest quartile) β	*p*	HGS (highest quartile) β	*p*	ASM/wt (highest quartile) β	*p*	HGS (highest quartile) β	*p*
Model 1	0.155	**0.006**	0.062	0.355	1.143	**0.005**	0.450	0.357	1.064	**0.002**	0.312	0.499
Model 2	0.159	**0.001**	0.055	0.403	1.173	**0.001**	0.394	0.404	1.067	**0.002**	0.308	0.507
Model 3	0.187	**0.002**	0.050	0.486	1.377	**0.001**	0.355	0.495	1.230	**0.005**	0.244	0.633
Model 4	0.190	**0.002**	0.050	0.485	1.396	**0.002**	0.355	0.493	1.249	**0.006**	0.244	0.632

	Total femur BMD				Total femur (T-score)				Total femur (Z-score)			
	ASM/wt (highest quartile) β	*p*	HGS (highest quartile) β	p	ASM/wt (highest quartile) β	*p*	HGS (highest quartile) β	*p*	ASM/wt (highest quartile) β	*p*	HGS (highest quartile) β	*p*
Model 1	0.132	0.067	− 0.007	0.926	1.053	0.064	− 0.041	0.950	1.007	0.066	− 0.164	0.800
Model 2	0.134	0.055	− 0.012	0.889	1.068	0.053	− 0.071	0.915	1.004	0.068	− 0.158	0.810
Model 3	0.181	**0.030**	− 0.009	0.923	1.419	**0.031**	− 0.058	0.937	1.355	**0.039**	− 0.153	0.835
Model 4	0.179	**0.026**	− 0.010	0.912	1.406	**0.027**	− 0.064	0.927	1.342	**0.035**	− 0.158	0.819

Generalized estimating equations.

*ASM/weight* appendicular skeletal mass adjusted weight, *HGS* handgrip strength.

Model 1: Crude analysis model (Bone mineral density and low muscle strength or low muscle mass), Model 2: Model 1 adjusted for age, Model 3: Model 2 adjusted for body mass index and body fat percentage, Model 4: Model 3 adjusted for HOMA-IR (homeostasis model assessment-insulin resistance).

Significant values are in bold.

#### Metabolic and inflammatory profile after bariatric surgery

Total cholesterol, LDL-cholesterol, triglycerides, fasting glucose, HbA1c, insulin, and HOMA-IR were reduced in both groups over the 1-year follow-up (*p* < 0.05, Table 5). In addition, HDL-cholesterol increased in females with and without sarcopenic-obesity parameters over the 1-year follow-up (*p* < 0.05, Table 5). No difference was found for the time x group interaction for these variables, except for HDL cholesterol, where females with SOP had increased HDL cholesterol during the 1-year follow-up compared to females without sarcopenic obesity parameters (I = 0.017, Table 5).

**Table 5:** Effects of bariatric surgery on biochemical variables in females with obesity and with sarcopenic-obesity parameters over a 1-year follow-up.

	Obesity (n = 21)				Sarcopenic-obesity parameters (SOP, n = 15)				*p*-value time	p-value group	*I*
	Baseline	3 months	6 months	1 year	Baseline	3 months	6 months	1 year			
Biochemical variables											
Total cholesterol (mg/dL)	176 ± 25	161 ± 30	164 ± 26	164 ± 29	194 ± 38	184 ± 29	182 ± 27	173 ± 24	0.023	0.050	0.373
HDL-cholesterol (mg/dL)	53 ± 10	46 ± 9	53 ± 10	56 ± 12	46 ± 9	47 ± 9	50 ± 9	56 ± 10	< 0.001	0.509	0.017
LDL-cholesterol (mg/dL)	99 ± 22	95 ± 27	92 ± 22	87 ± 25	116 ± 32	107 ± 25	111 ± 24	93 ± 24	0.004	0.059	0.096
Triglycerides (mg/dL)	128 ± 85	93 ± 28	93 ± 42	88 ± 56	152 ± 97	115 ± 50	102 ± 35	101 ± 45	0.017	0.317	0.651
Insulin (µU/mL)	18 ± 13	7 ± 3	6 ± 3	5 ± 3	18 ± 8	9 ± 5	7 ± 5	7 ± 5	< 0.001	0.398	0.389
Fasting glucose (mg/dL)	91 ± 22	82 ± 5	80 ± 6	79 ± 6	95 ± 18	84 ± 8	81 ± 10	78 ± 9	0.001	0.627	0.619
HOMA-IR	3.8 ± 2.0	1.8 ± 1.3	1.6 ± 1.2	1.5 ± 1.4	4.6 ± 2.6	2.0 ± 1.4	1.5 ± 1.4	1.6 ± 1.4	< 0.001	0.573	0.669
HbA1c (%)	5.9 ± 0.9	5.4 ± 0.3	5.3 ± 0.4	5.3 ± 0.4	6.2 ± 0.7	5.7 ± 0.4	5.5 ± 0.3	5.5 ± 0.5	< 0.001	0.148	0.456
hs-CRP (mg/dL)	6.0 ± 5.3	3.4 ± 3.1	3.1 ± 3.4	2.6 ± 4.2	9.1 ± 12.0	5.2 ± 6.3	4.6 ± 5.8	1.5 ± 1.3	0.008	0.450	0.286

Mixed between-within subjects ANOVA, Data are expressed as mean ± SD.

*HDL- cholesterol* high density lipoprotein cholesterol, *LDL-cholesterol* low density lipoprotein cholesterol, *HOMA-IR* homeostasis model assessment—insulin resistance, *HbA1c* glycated hemoglobin, *hs-CRP* high-sensitive c-reactive protein.

## Discussion

The results of this study showed that the percentage of weight loss, fat mass and body fat percentage, ASM/wt, gait speed, muscle mass and strength when properly analyzed divided by body size, and biochemical variables (glycemic, lipid, and inflammatory) were improved in females with and without sarcopenic-obesity parameters over a 1-year follow-up of bariatric surgery. In addition, the study demonstrated for the first time that females with sarcopenic-obesity parameters had lower BMD in L1-L4 and femoral neck in the preoperative period, both groups decreased BMD over time, and ASM/wt was positively associated with BMD over a 1-year follow-up of bariatric surgery.

Sarcopenia is a common disease in the elderly; however, young subjects with obesity may exhibit sarcopenia due to excessive weight gain, adipocyte hypertrophy, ectopic fat deposition in the muscle, inflammation, and insulin resistance^22^. In addition, a history of recent weight loss (including voluntary weight loss and a long-term restrictive diets), physical inactivity, and bariatric surgery may contribute to skeletal muscle mass loss^22,23^.

Low muscle mass has been reported in females with obesity and is associated with low HGS and BMD^5^. The present study evaluated parameters of sarcopenic obesity and not the diagnosis of sarcopenic obesity in middle-aged females who underwent bariatric surgery. There are several reasons for this: first, muscle mass and muscle function do not have the same clinical relevance during the aging process^24^; second, muscle strength and muscle mass are not congruent, i.e. muscle strength can decrease even if muscle mass is maintained or increased^25^; and lastly, there is no international consensus on a definition of sarcopenia^26^ and no clinical and research guidelines specific to Brazil. Therefore, it is reasonable to suggest that muscle mass and muscle strength need to be defined independently because they may have different clinical implications in middle-aged females.

It has been suggested that weight loss promoted by bariatric surgery results in changes in body composition with loss of fat mass, but also loss of skeletal muscle mass and bone mass^6,7,27–29^. The present study showed a significant decrease in weight, body mass index, total fat mass, body fat percentage, total skeletal muscle mass, fat-free mass, fat-free mass index and HGS in both groups over the 1-year follow-up period. However, when muscle mass and strength were analyzed relative to body size rather than in absolute terms, an improvement after bariatric surgery was found, suggesting that the assessment of absolute muscle mass and strength after surgery should be used with caution and that the adjusted assessment may be better applied.

Studies are needed to better understand the clinical implications of the loss of skeletal muscle mass that occurs after bariatric surgery. One of these gaps is the assessment of absolute skeletal muscle mass rather than relative skeletal muscle mass loss^30,31^. In the current study, there was a loss of total skeletal muscle mass during the follow-up, but when considering the ASM/wt, there was an increase in relative muscle mass. These data are consistent with a previous study that reported an improvement in the proportion of fat mass to muscle mass in the group that lost more than 50% of excess weight, despite a decrease in absolute muscle mass^32^.

In the present study, although there was a decrease in absolute HGS, an increase in HGS adjusted for body mass index was observed in both groups during a 1-year follow-up period. Similarly, a prospective cohort showed a 9% decrease in absolute muscle strength and a 32% increase in HGS adjusted for body mass index in the 12 months after Roux-in-Y gastric bypass surgery^33^. Here, we have demonstrated that females with SOP had lower HGS and HGS adjusted for body mass index than females without SOP because this is part of the criteria for group definition. However, both groups showed a decrease in HGS and an increase in HGS adjusted for body mass index during 1 year of follow-up. Whether strength training before and after bariatric surgery can have beneficial effects on HGS in SOP patients remains to be determined.

Worldwide guidelines for bariatric surgery have recommended that the cardiovascular risk profile of patients with obesity must be assessed prior to surgery. However, there is no formal recommendation for bone and muscle assessment before a bariatric surgery procedure^34,35^. It is already documented that bariatric surgery, sarcopenia, and obesity increase the risk of bone compromise and bone fracture^10,28,29,36^. Although the negative repercussions of sarcopenic obesity on bone are already recognized, to our knowledge, no studies have assessed the association between sarcopenic obesity parameters and bariatric surgery. In the current study, BMD was reduced at all sites (L1-L4, femoral neck, and total femur), as well as Z-score and T-score in females with and without sarcopenic-obesity parameters.

Comparing the two groups during a 1-year follow-up, females with SOP had lower BMD, Z-score, and T-score in the L1-L4 and femoral neck than the obesity group. This data is important considering that bariatric surgery increases the risk of bone fracture during follow-up due to nutritional factors (low calcium intake and vitamin D deficiency), hormonal factors (decreased estrogen, leptin, insulin, amylin, and increased parathyroid hormone), and bone architecture changes^37^. Fracture risk appears to be higher after two to five years of bariatric surgery and after Roux-in-Y gastric bypass than sleeve gastrectomy^28,29^. Furthermore, it is important to emphasize that not only does the presence of metabolic factors increase mortality, but osteoporosis and fractures are also risk factors for higher mortality^38,39^. In fact, whether sarcopenic obesity or sarcopenic-obesity parameters before bariatric surgery increases bone fracture and higher mortality remains to be elucidated.

In the present study, females with obesity from both groups displayed a decrease in total cholesterol, LDL-c, triglycerides, fasting glucose, HbA1c, insulin, HOMA-IR, and an increase in HDL-cholesterol over the 1-year follow-up. This finding corroborates with early studies reporting metabolic improvement after bariatric surgery^40,41^. However, there were no differences between the two groups regarding metabolic and inflammatory profiles.

**Figure 1:**
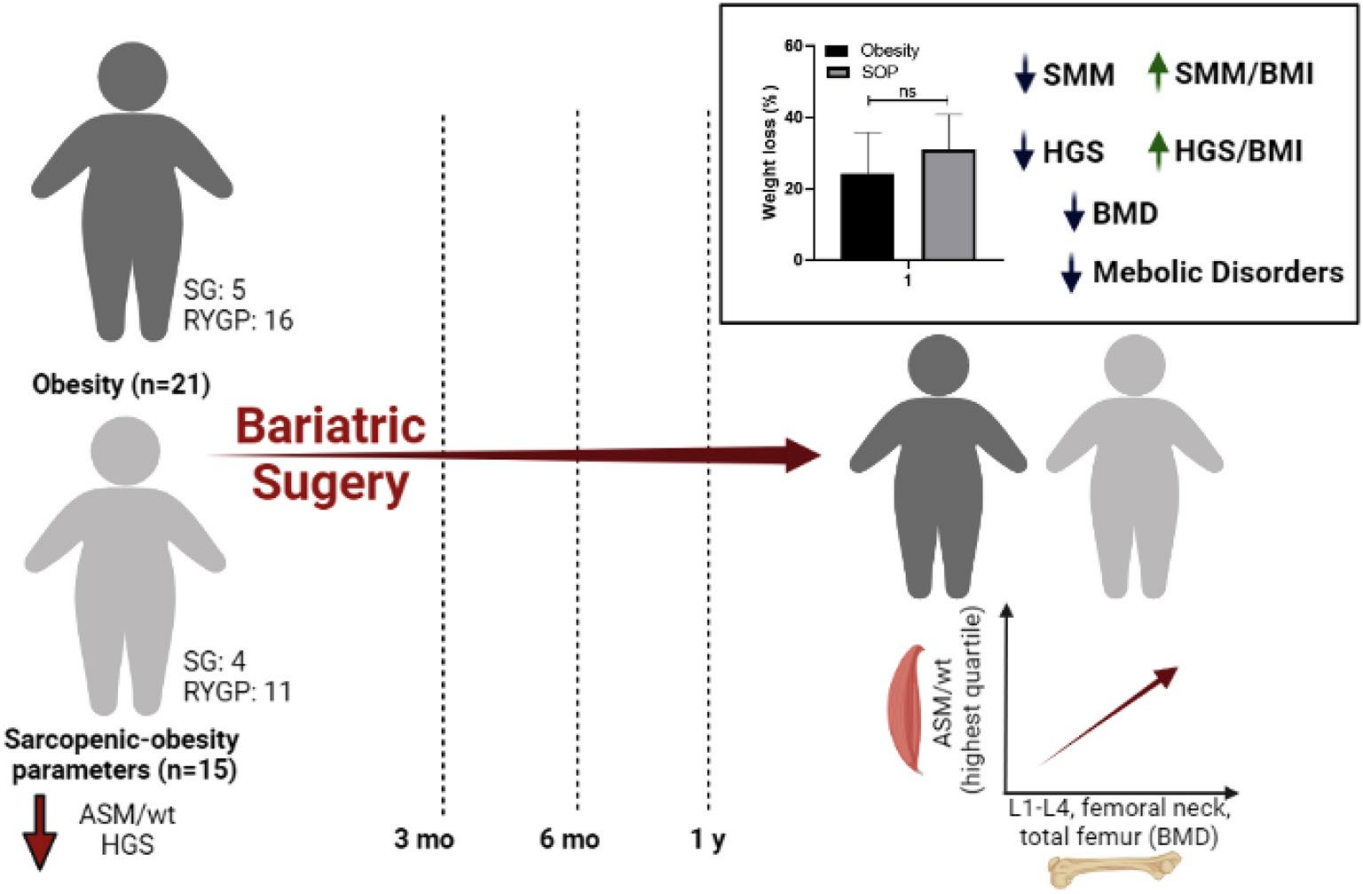
Effects of preoperative sarcopenia-related parameters on the musculoskeletal and metabolic outcomes after bariatric surgery. Females with preserved muscle mass and strength or females with sarcopenia-related parameters had reduced metabolic disorders and decreased bone mineral density during a 1-year follow-up after bariatric surgery. In addition, the findings showed that although muscle mass and strength have decreased over a 1-year follow-up, there was an improvement in the muscle mass and strength when analyzed relative to body size. Lastly, the findings showed the highest quartile of ASM/wt was positively associated with BMD variables in a longitudinal analysis. *ASM/weight* appendicular skeletal mass adjusted weight, *HGS* handgrip strength, *SMM* skeletal muscle mass, *BMD* bone mineral density, *BMI* body mass index.

Given that muscle mass or strength may affect BMD, a prospective analysis of the association was performed to answer this gap. The findings showed that females with the highest quartile of ASM/wt had a positive association with BMD. Muscle mass and muscle strength should both be assessed in pre-surgery in middle-aged females, since the proper diagnosis of sarcopenic obesity requires appropriate follow-up by a multidisciplinary health care team. The results of this study are summarized in Fig. 1.

The number of participants is a limitation of the study. The COVID-19 pandemic stopped bariatric surgery in several hospital and clinics. Our sample consisted of females, and extrapolating these results to males would not be appropriate. We evaluated parameters of sarcopenic obesity and not the diagnosis of sarcopenic obesity, which could interfere with the results. Unfortunately, we have a lot of heterogeneity in determining cutoff points for low ASM/wt and HGS. We do not have a formal recommendation for low ASM/wt and HGS in middle-aged individuals. However, our study brings the relevance of the association of ASM/wt and HGS variables with BMD outcomes. Studies that include not only bone mass but also bone quality and metabolism would be needed.

Despite the limitations, to our knowledge, this was the first study to evaluate individuals with parameters related to sarcopenic obesity and their clinical responses during follow-up. Furthermore, this current study suggests that a better musculoskeletal stratification should be performed before bariatric surgery to identify individuals with a greater propensity to lose bone mass during the follow-up of this surgery, thus promoting a better clinical management of these cases.

## Conclusion

Bariatric surgery promoted weight loss, improved body fat percentage, and improved glucose, lipid, and inflammatory marker in females with and without sarcopenic-obesity parameters. Although skeletal muscle mass and HGS decreased throughout the follow-up, there was an improvement in the muscle mass and strength when analyzed relative to body size. The highest quartile of ASM/wt was positively associated with BMD variables in a longitudinal analysis, suggesting that preserved ASM/wt in pre-surgery may be beneficial for BMD after 1 year of bariatric surgery.

## Data Availability

The data that support the findings of this study are available from the corresponding author upon reasonable request.

## Abbreviations

ASM: Appendicular skeletal mass
ASM/wt: Appendicular skeletal mass adjusted for weight
BMD: Bone mineral density
hs-CRP: High sensitivity C-reactive protein
HGS: Handgrip strength
HbA1c: Glycated hemoglobin
HDL-cholesterol: High-density lipoprotein cholesterol
HOMA-IR: Homeostasis model assessment-insulin resistance
LDL-cholesterol: Low-density lipoprotein cholesterol
